# The versatile role of exosomes in human retroviral infections: from immunopathogenesis to clinical application

**DOI:** 10.1186/s13578-021-00537-0

**Published:** 2021-01-15

**Authors:** Jafar Rezaie, Cynthia Aslan, Mahdi Ahmadi, Naime Majidi Zolbanin, Fatah Kashanchi, Reza Jafari

**Affiliations:** 1 Solid Tumor Research Center, Cellular and Molecular Medicine Research Institute, Urmia University of Medical Sciences, Shafa St, Ershad Blvd., P.O. Box: 1138, 57147 Urmia, Iran; 2 Immunology Research Center, Tabriz University of Medical Sciences, Tabriz, Iran; 3 Department of Immunology, Faculty of Medicine, Tabriz University of Medical Sciences, Tabriz, Iran; 4 Tuberculosis and Lung Diseases Research Center, Tabriz University of Medical Sciences, Tabriz, Iran; 5 Department of Pharmacology and Toxicology, School of Pharmacy, Urmia University of Medical Sciences, Urmia, Iran; 6 School of Systems Biology, Laboratory of Molecular Virology, George Mason University, Discovery Hall Room 182, 10900 University Blvd., Manassas, VA 20110 USA

**Keywords:** Exosomes, Extracellular vesicles, HIV-1, HTLV-1, Retroviruses

## Abstract

Eukaryotic cells produce extracellular vesicles (EVs) mediating intercellular communication. These vesicles encompass many bio-molecules such as proteins, nucleic acids, and lipids that are transported between cells and regulate pathophysiological actions in the recipient cell. Exosomes originate from multivesicular bodies inside cells and microvesicles shed from the plasma membrane and participate in various pathological conditions. Retroviruses such as Human Immunodeficiency Virus -type 1 (HIV-1) and Human T-cell leukemia virus (HTLV)-1 engage exosomes for spreading and infection. Exosomes from virus-infected cells transfer viral components such as miRNAs and proteins that promote infection and inflammation. Additionally, these exosomes deliver virus receptors to target cells that make them susceptible to virus entry. HIV-1 infected cells release exosomes that contribute to the pathogenesis including neurological disorders and malignancy. Exosomes can also potentially carry out as a modern approach for the development of HIV-1 and HTLV-1 vaccines. Furthermore, as exosomes are present in most biological fluids, they hold the supreme capacity for clinical usage in the early diagnosis and prognosis of viral infection and associated diseases. Our current knowledge of exosomes' role from virus-infected cells may provide an avenue for efficient retroviruses associated with disease prevention. However, the exact mechanism involved in retroviruses infection/ inflammation remains elusive and related exosomes research will shed light on the mechanisms of pathogenesis.

## Background

Apart from the secretion of conventional vesicles containing hormones or neurotransmitters by specialized cells, all mammalian cells release double-phospholipids vesicles known as extracellular vesicles (EVs) [1, 2]. Initially, the secretion of EVs has been considered a way to eliminated unwanted molecules from cells [3], however, according to intensive recent studies, these vesicles are more than waste transporters and they are considered cell-to-cell communication mediators by transferring different types of components including nucleic acids, proteins, and lipids, and participate in normal cellular homeostatic processes or pathological progression [4, 5]. EVs are present in different body fluids including plasma, breast milk, urine, cerebrospinal fluid (CSF), bile, bronchoalveolar lavage fluid, peritoneum, saliva, and semen [6, 7]. Although the term EVs refers to this kind of vesicles, they are heterogeneous in size, shape, and even cargo. Regarding the International Society for Extracellular Vesicles (ISEV) guidelines, EVs are broadly divided into three main categories based on their size and origin: apoptotic bodies, microvesicles (MVs), and exosomes. Apoptotic bodies are 1–6 µm EVs deriving from apoptotic cells. MVs range in size of 100–500 nm in diameter, are produced by outward budding of the plasma membrane of cells, releasing into the extracellular matrix. Exosomes are 30–150 nm EVs originating from multivesicular bodies (MVBs), which are transitional vesicles inside the endosomal system, and are released into the extracellular milieu upon the fusion of MVBs with the cell membrane. EVs can reach neighboring and distant target cells, and affect their function and fate. Besides, EVs have been shown to play pivotal roles in the pathogenesis of different diseases such as cancer and infection diseases [8]. EVs produced by infected cells can carry virus-related particles that trigger an infection in recipient cells and immunomodulatory responses in the host [9–11]. Understanding the molecular mechanisms regulating the virus entry, duplication, spreading, and infection of such Retroviruses as Human Immunodeficiency Virus -type 1 (HIV-1) and Human T-cell leukemia virus (HTLV)-1 may provide us with a tool to design new approaches for diagnosis and treatment. As EVs participate in pathogenesis of viruses and they may be a promising tool for the treatment of virus infection, we summarize the existing research on EVs in HIV-1 and HTLV-1 infection. Therefore, we describe exosomes biogenesis, secretion, isolation and their key roles in HIV-1 and HTLV-1 infection. Further, we focus on clinical application of exosomes in HIV-1 associated diseases.

### Biogenesis of exosomes

**Fig. 1:**
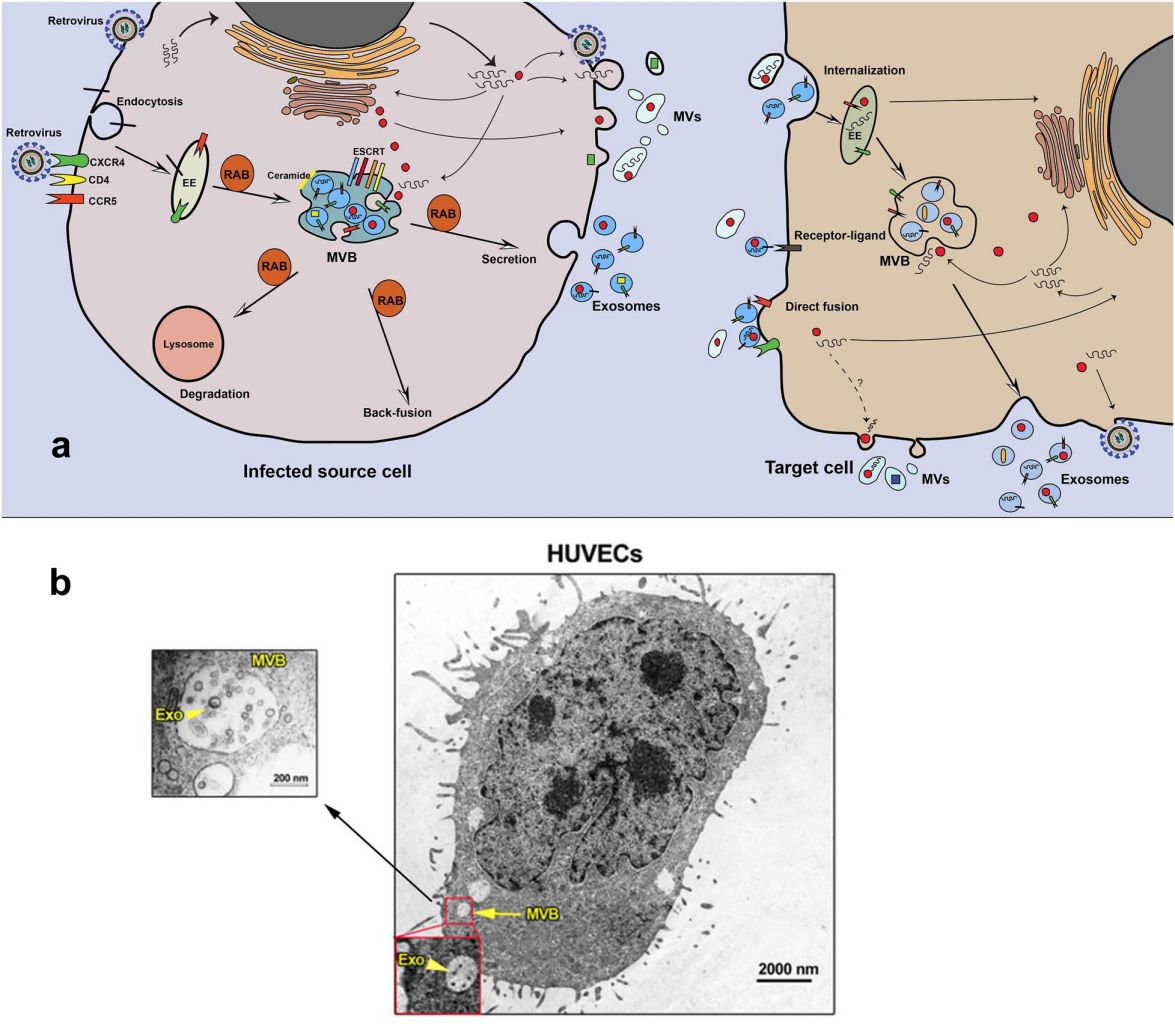
Biogenesis, trafficking, and uptake of exosomes from virus-infected cells. Retrovirus can use the exosomal secretory pathway to increase infection in target cells (**a**). TEM micrographs of multivesicular bodies and exosomes in HUVECs (License number: 4916980066066) (**b**) (a). EE: early endosome; Exo: exosome; HUVECs: Human umbilical vein endothelial cells; MVB: multivesicular body; MVs: microvesicles

**Fig. 2:**
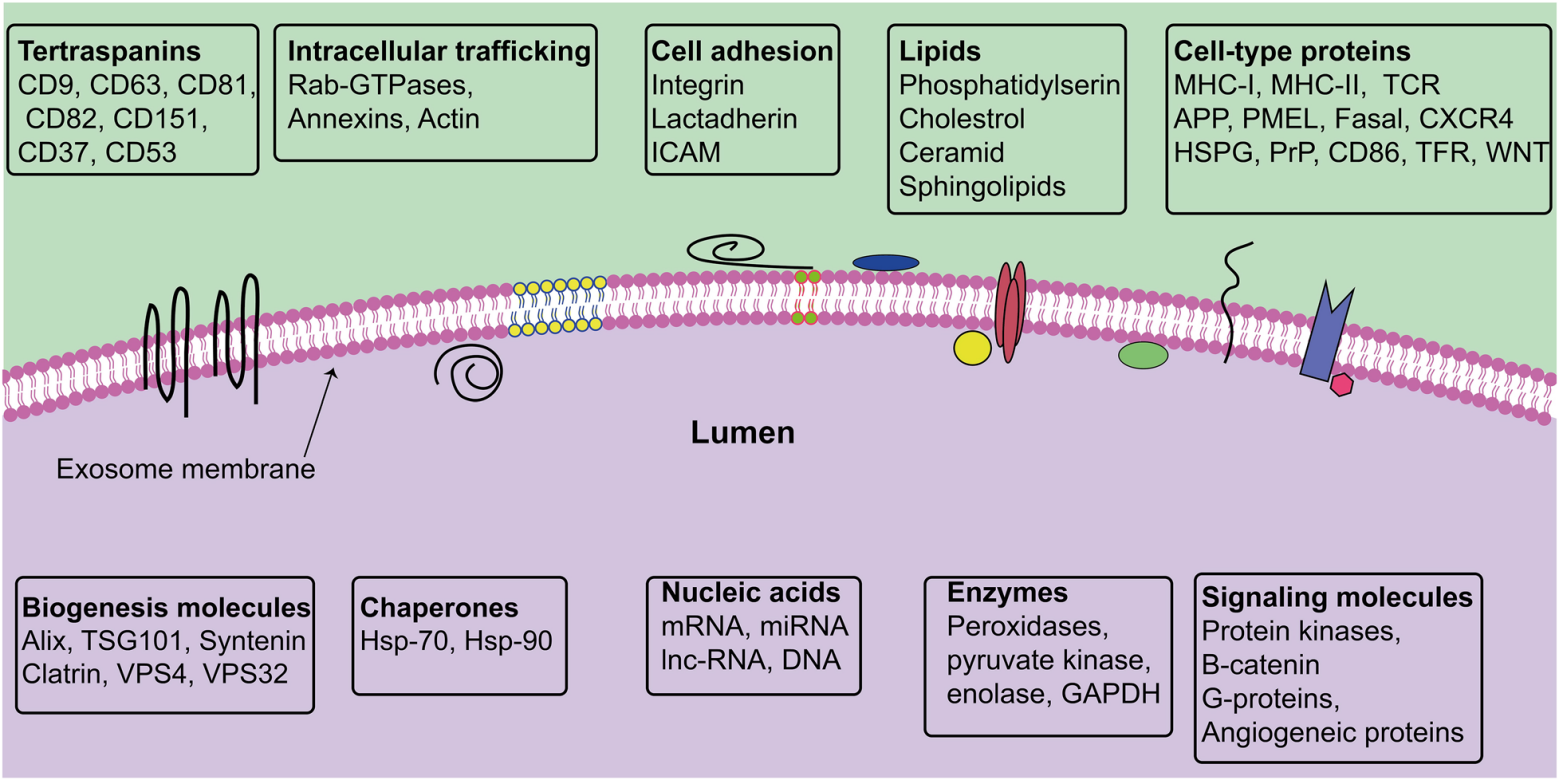
Exosomes contain different types of components both on their surface and lumen and this content can vary widely between cells and conditions

Exosomes and MVs represent a unique way of generation; indeed, exosomes are generated from the inward budding of MVBs membrane as intraluminal vesicles (ILVs) and secreted into the extracellular matrix upon MVBs and the plasma membrane fusion [12] (Fig. 1, Table 1). Different molecules and pieces of machinery are involved in exosome biogenesis [6, 12]. These types of machinery segregate cargo on microdomains of MVBs membrane and subsequently invagination and fission of nascent ILVs containing sequestered cytosol (Fig. 1). In this regard, ESCRT machinery has been shown to drive sorting, membrane shaping, and abscission during exosome formation. The ESCRT machinery is a collection of different ESCRT complexes with stepwise mediated exosome biogenesis wherein ESCRT-0 identifies ubiquitylated transmembrane cargo and cluster them on microdomains incorporation with ESCRT-I. Next, ESCRT-II and ESCRT-III induce the invagination of MVBs membrane and fission of ILVs [13, 14]. ALG-2 interacting protein X (ALIX) is an accessory protein that facilitates the abscission of ILVs into the lumen of MVBs in an ATP-consuming manner. Several microdomains participate in sorting soluble components, like cytosolic proteins and RNA molecules into MVBs/ILV [13, 14]. Some components of ESCRT machinery are secreted within exosomes and may serve as exosome markers including Alix and HRS. However, cargo clustering, loading, and MVBs membrane invagination can take place by ESCRT-independent mechanisms. It was reported that Ceramide is involved in exosome biogenesis [15, 16]. Ceramide, a cone-shaped lipid, is generated from sphingomyelin by the activity of neutral type II sphingomyelinase and mediates the formation of membrane subdomains, which induces inward budding of MVBs membrane [17]. Furthermore, ceramide may be metabolized into sphingosine-1 phosphate, which binds to its receptor coupled with the G protein, in turn, this interaction contributes to exosome loading [18]. Besides, tetraspanin proteins such as CD63 [19], CD9, and CD81, CD82 [20], and other components like syndecan-syntenin-ALIX complex [21, 22], VCAM-1 and α4 integrin [23, 24], and phosphatidic acid (PA) [25] are involved in sorting different molecules into exosomes. Sorting of some molecules may be the result of together with exosomal and membrane cargo. For instance, cytosolic protein may co-directed into exosomes with the heat shock cognate 71 kDa protein (HSC70) and chaperones heat shock 70 kDa protein (HSP70) [26, 27]. Similarly, Glycosylphosphatidylinositol (GPI)-anchored proteins, the membrane-associated proteins, are sorted into exosomes due to their affinity for lipid rafts, which are enriched in MVBs membrane [28]. Exosome cargo can be attained through the plasma membrane, the Golgi apparatus, and also the cytoplasm. Thus, exosomes contain various types of components on their surface and lumen (Fig. 2). However, exosomes are heterogeneous in size and component and it may be possible that such exosome subpopulations can generated from MVBs subpopulations [29, 30]. It was suggested that the type of sorting/biogenesis machinery may determine the destination of MVBs among exosome secretion and lysosomal degradation pathways. As shown in Fig. 1, MVBs inside cells follow three possible fates including secretion; degradation, and back-fusion. Intracellular trafficking of MVBs is mediated by cytoskeleton (microtubules and actin), molecular motors (kinesins, dynein, and myosins), and different types of Rab proteins [6, 12]. It is apparent that the composition of MVBs/ILVs and type of recruited sorting machineries and also cell condition determine the target of MVBs. In the final step, SNARE proteins and Rab proteins mediate the fusion of MVBs with the plasma membrane to release ILVs as exosomes [31]. Although different cells release exosomes with distinct molecules, however, exosomes contain the conventional markers which make them distinctly different from other EVs as CD63, CD81, CD82, CD9, TSG101, and also ALIX [32].

**Table 1:** Extracellular vesicles characteristics

Extracellular vesicles	Other names	Origin	Size (nm)	Markers
Exosomes	Exosome-like vesicles	Multivesicles bodies	30–150	CD29, CD63, CD81, CD82, CD9
	Dexosomes			
	Nanovesicles			
	Prostasomes			
	Tolerosomes			
Microvesicles	Microparticles	The plasma membrane	100–1000	Annexin V, Flotinin-2,
	Shedding vesicles			
	Blebbing vesicles			CD40, CD62, Integrins
	Oncosomes Migrasomes			
	ARRMs			
	Neurospheres			
Apoptotic bodies	Microparticles	Apoptotic bodies	1000–6000	Annexin V, DNA, Histones

### Biogenesis of microvesicles

As described above, MVs are another EVs subclass that originate directly from the plasma membrane known as shedding vesicles. It has been shown that MVs generation is depended on stimuli, but exosomes may be generated constitutively or following a stimulus [33]. The generation of MVs resembles a virus outward process from infected cells. This process is complex and comprises several reorganizations inside the plasma membrane, such as alterations in protein and lipid portions, and Ca2^+^ concentration [34]. In this scenario, different Ca2^+^-dependent enzymes such as scramblases, calpain, aminophospholipid translocases (floppases and flippases) mediate reorganizations in the asymmetry of membrane phospholipids leafs (translocation of phosphatidylserine from the inner leaflet to outer one), which subsequently induces the membrane blend and rearrangement of the actin cytoskeleton, which in turn, promotes membrane outside budding and shedding of MVs [35, 36]. Cholesterol plays a key role in MVs generation [37]. Rho-associated protein kinases (ROCK) is another molecule that contributes to forming MVs has been reported. For instance, Li et al. using breast and ovarian cancer cell lines showed that inhibition of the activity of ROCK1 and ROCK2 decreased MVs formation [38]. In the downstream pathway, ROCK1 and ROCK2 can activate LIM kinase (LIMK) and myosin light chain kinase (MYLK), which phosphorylates cofilin and myosin respectively, thus inducing filaments contraction and actin polymerization filaments toward the cytoplasm cortex and the plasma membrane. In keeping, an ‘actin-ring’ complex is shaped, which is necessary for the shedding of MVs [38]. Additionally, there exists some evidence ESCRT-I and ESCRT-III complexes contribute to MVs generation [39, 40]. Similar to exosomes loading, proteins, and lipids and of the membrane are directed to sites of MVs budding as a result of their affinity for lipid rafts or anchored to the plasma membrane lipids including the oligomeric cytoplasmic proteins [41, 42]. The detailed mechanisms involved in nucleic acids sorting is still largely uncertain, however, one possible mechanism is that the conserved zipcode RNA sequence motifs in the 3ʹ untranslated regions in mRNA may participate in sorting RNA into MVs [43].

### EVs uptake routes

Once secreted into the extracellular milieu, EVs can affect target cells and deliver their cargo to drive signaling pathways and cause functional and phenotypic changes, affecting their pathophysiologic condition [44]. The way of EVs interact with the cell membrane and the mechanisms involved in the transferring of EVs cargo is not fully understood. However, there needs to be docking at the plasma membrane of target cells, followed by vesicle internalization, the activation of surface receptor-ligand interaction, or direct fusion with the target cell membrane [44, 45] (Fig. 1). Vesicle internalization may comprise different mechanisms as endocytosis, pinocytosis, and phagocytosis. In receptor-ligand interaction, molecules located on EVs surface interact with corresponding molecules on the target cell surface. For example, tetraspanins on the exosomes interact with integrins of the cell membrane [46, 47], and also integrins on the exosomal membrane interact with adhesion molecules like intercellular adhesion molecules (ICAMs) on the target cell membrane and induce cell signaling [48]. In direct fusion way, EVs membrane fuse with target cell membrane like a conventional membrane fusion process by which EVs cargo are discharged into the cytoplasm of target cells. Taken together, mechanisms behind EVs uptake are multifaceted and probably depend on the type of EVs and recipient cells, and they may be associated with the downstream effects and signaling pathways facilitated by EVs [45]. Finally, in a single cell, it is not clear that these pathways work synergically or independently in regulating cargo delivery.

### Exosome isolation methods

Exosomes have 30–150 nm size in diameter with a density in sucrose of 1.13–1.19 g/ml [49]. Exosomes appear cup-shaped and the spherical particles using transmission electron microscopy and the cryoEM technique respectively [50]. Exosomes from different sources contain various proteins and lipids both on the surface and lumen with different sedimentation characteristics (Fig. 2). For example, adipose tissue-derived exosomes encompass high lipid content and require modification in their isolation methods [51], and for exosome isolation from cultured media, it is necessary to use either exosome-free fetal bovine serum (FBS) or FBS-free media. Exosomes can be found in all body fluids and different methods have been used to isolate exosomes from different bio-fluids. Moreover, the purification of EVs (exosomes) and the virus is a quickly progressing field. Viruses and EVs/exosomes share several similarities including radii, densities, biogenesis, maturation, and the capacity to serve as delivery systems for nucleic acids [52–54]. Regarding several overlapping properties, isolation of exosomes from viruses face different challenges. According to literature, among several methods, two methods including nanoFACS and immunological separation are suitable for separating exosomes from viruses in infected samples [55]. However, these methods may have some disadvantages including saturation of epitope binding beads can lead to product and function loss or only certain flow cytometers calibrated for nanoFACS analysis [55, 56]. Different methods used to isolate exosomes/viruses include centrifugation, filtration, chromatography, polymer-based precipitation, and immunological separation, nanoFACS [31, 44]. In Table 2, we summarized methods used to isolate exosomes and described the pros and cons of exosomes/virus isolation methods.

**Table 2:** Methods of exosome isolation

Isolation methods	Mechanism	Advantages	Disadvantages
Ultracentifugation	The method consists of a series of centrifugation to remove cells and debris and precipitate exosomes based on size and density	This method is the standard and gives pure exosomes and viruses from bio-fluids and cell culture media	The efficiency of the method is low for the isolation of exosomes from plasma and serum. Overlapping densities between viruses and exosomes
Density gradient centrifugation	This method is ultracentrifugation aimed at an iodixanol or sucrose based on a density gradient	Through this method small and low-density exosomes are isolated from particles, other vesicles, and contaminants	Sensitive to the centrifugation time and needs more minuteness. Overlapping densities between viruses and exosomes
Filtration	This method uses ultrafiltration membranes to isolate exosomes from proteins aggregation and other macromolecules	In this method, small particles and soluble molecules separate from exosomes. Exosomes are concentrated on the filtration membrane	Exosomes may be lost due to adhering to the filtration membranes. Besides, the additional force may be deformed or damaged exosomes. Only useful when starting with large volumes Overlapping densities and size between viruses and exosomes
Size exclusion chromatography	In this method, using a column packed with porous polymeric beads and size-exclusion chromatography macromolecules are separated based on their size. It applies	In this method, large and small molecules are separated. This method can separate exosomes from viruses. Also, the structure of isolated exosomes is not changed by the shearing force	The method is time-consuming. Overlapping size between viruses and exosomes
Immunological separation	Several immunological methods such as Magnetic beads bound to the specific antibodies and the ELISA-based separation method	This method selectively isolates exosomes or subpopulation of exosomes. This method can separate exosomes from viruses Also, it is applicable for the characterization and quantitation of exosomal markers	Small sample volumes are required and the isolated vesicles may fail the functional activity as well as can require initial concentration step
Polymer-based precipitation	This method which is prepared as commercial kits comprises mixing the biofluids with precipitation solution, incubation step, and low-speed centrifugation	This method has a mild effect on isolated exosomes and the usage of neutral pH	In this method, exosomes are isolated with contamination and the presence of the polymer material may affect downstream analysis. Overlapping size between viruses and exosomes
Isolation by sieving	In this method, exosomes are isolated by sieving through a membrane and filtration by electrophoresis or pressure	Fairly short isolation time and isolated exosomes are pure	Low recovery of isolated exosomes. Overlapping size between viruses and exosomes
Microfluidics-based techniques	This method is microscale isolation based on exosomes immunoaffinity, size, and density	This method is a low cost, fast, portable, and high portability	Limitation in standardization and large scale tests on clinical samples, Limitation in method validation, moderate to low sample capacity. Overlapping size between viruses and exosomes
nanoFACS	Similar to fluorescence assisted cell sorting (FACS), nanoscale flow cytometry and nanoFACS are meant to identify and sort EVs subpopulations based on a heterogeneous input population	This method can separate exosomes from viruses based on indirect fluorescence labeling or de novo labeled proteins (like GFP-HSV-1 fusion proteins, and GFP-Gag). It can be employed to rapidly characterize heterogeneous input mixtures without the need to concentrate them first	Only certain flow cytometers calibrated for nanoFACS Sample typically requests to be diluted earlier nanoFACS and may be additional diluted post sorting

### Extracellular vesicles and virus

EVs naturally are bio-container for delivering different bio-molecules between cells. A growing body of evidence indicates such EVs as MVs and exosomes released from virus-infected cells transfer virus components like proteins, genomic molecules [57], as well as virus receptors to recipient cells, consequently, making them more vulnerable to infection (Fig. 1). Virus-infected cells release more MVs as compared to non-infected cells. Besides, the component of these MVs is different from those released by non-infected cells that promote pathological condition [58]. MVs released by virus-infected cells may serve as a biomarker for virus infection. For example, during HIV infection, platelets abundantly release MVs containing mitochondria, which are different from those of healthy cells; suggesting biomarker for HIV infection [59]. During virus infection, the shedding of MVs containing viral proteins and glycoproteins, can make surrounding cells susceptible to infection and reduce immune responses [60, 61]. There is evidence that common ways are involved in virus lifecycle and exosome formation and uptake. For instance, ESCRT machinery mediates EVs (exosomes and MVs) and virus formation and the mode of EVs and virus uptake is somewhat similar. Moreover, infected cells release EVs (exosomes and MVs) with distinct cargo, which differs from healthy cells, virus infection alters EVs loading and biogenesis mechanism independent of viral proteins/nucleic acids. Therefore, these new modified EVs probably also change the immune response of the host (Table 3).

**Table 3:** Exosomal cargoes in retroviral infection

Exosome cargo		Origin of exosome	Target cell	Function	References
Protein	Nef	HIV-1 infected cells	T cells	Making the latent cells more vulnerable to HIV infection	[72]
		T cells	T cells	Inhibiting the generation of CD4 + EVs from T cells	[73]
		Macrophages	T cells	Inhibiting T cell function via beta-COP-dependent pathway and degradation of MHC-I and CD4 + molecules	[76]
		HIV-1-infected macrophages	B cells	Inhibiting the adaptive immune response by deterring the IgA and IgG production in B lymphocytes	[77]
		HIV-1 infected cells	Macrophages	Increasing secretion of pro-inflammatory cytokines	[91]
		Plasma from patients with HAD	SH-SY5Y neuroblastoma cells	HAND progression	[98]
		HIV-infected microglia	Microglia	HIV-induced neuropathogenesis	[99]
	CCR5	PBMNCs and CCR5 + ovary cells	CCR5-null cells	Enhancing HIV-1 infection	[78]
	CXCR4	Megakaryocytes and platelets	CXCR4-null cell	Enhancing HIV-1 infection	[79]
	APOBEC3G	HIV-infected cells	PBMCs	Preventing virus production	[82]
	cGAMP	HIV-infected cells	DCs	Interferon upregulation	[83, 84]
	Tat and TAR	CSF samples of HIV-positive individuals	Not Assigned	Inducing pro-inflammatory responses	[87]
	Tat	Tat-expressing primary astrocytes	SHSY-5Y	Neurite reduction and neuron death	[97]
	Notch4 and oxidative stress markers	Plasma of HIV + patients	THP-1 monocyte cells	Inducing pro-inflammatory responses	[92]
	Fibronectin and galectin-3	HIV-1 infected DCs	T-cells	Up-regulating the pro-inflammatory cytokines expression	[93]
	Tax	HTLV-1-infected cells	Microglia cells	Inducing the production of proinflammatory cytokines	[132]
		HAM/TSP patients PBMCs and CSF samples	Uninfected PBMCs	Lessening the CD4 + CD25 + T cells	[133]
mRNAs & miRs	TAR	HIV-infected cells	293 T cells	Protecting cells from apoptosis for production of virus in infected cells	[80]
		HIV-infected cells	Primary macrophages or mouse neuronal cells	Stimulating the secretion of IL-6 and TNF-α, the pro-inflammatory cytokines	[88]
		HIV-infected T cells	HSC3 HNSCC and H1299 lung cancer cells	Development of lung cancer and HNSCC	[102]
	vmiR88 and vmiR99	HIV-infected macrophages and the sera of HIV + individuals	THP-1 macrophages	Inducing an inflammatory response	[85]
	miRNA-29b	Astrocytes treated with both morphine and HIV Tat	Neurons	Downregulation of PDGF-B expression and neuronal cell death	[94]
	miRNA-155-5p	HIV-infected T cells	Cervical cancer cells	Up-regulating the expression of cytokines such as IL-1, IL-6, and IL-8	[100]
	Tax and HBZ	HTLV-1-infected cells	PBMCs	Infection progression	[128]

### Exosomes and HIV-1

#### HIV-1

HIV-1 belongs to the Lentivirus genus of the Retroviridae family and is currently classified into two types, HIV-type 1 (HIV-1) which is more common, and HIV-type 2 (HIV-2) [62]. The HIV-1 genome is composed of roughly 9 kilobases in length, which encodes 9 genes and 15 proteins [63]. HIV-1 weakens the patients' defense system via targeting and destroying immune cells that express CD4 receptor and either the CCR5 or the CXCR4 co-receptors, especially CD4^+^ T cells, leading to immunodeficiency in the last stage of disease [64]. HIV/AIDS disease development comprises an acute stage, after the primary infection, characterized by a high viral load which greatly increases transmissibility and a substantial decline of host CD4^+^ T cell counts, which can lead to the influenza-like syndrome. Following acute infection, there is an asymptomatic phase with some CD4^+^ T cell recovery and a decline of viral RNA, however, later developing into a continuous decline of CD4^+^ T cells and an increase of viral RNA, indicating the chronic phase of infection. This phase may last over 10 years before individual advances to the last stage of HIV infection, acquired immunodeficiency syndrome (AIDS), though it may advance faster in some people [64]. AIDS is defined as the most severe stage of HIV infection, diagnosed with a CD4^+^ T cell count of fewer than 200 cells/mm^3^ [65]. HIV-1 infection promotes systemic diminution of CD4^+^T cells, which, in turn, results in impaired cell-mediated immunity, a wide spectrum of cancers, and increases susceptibility to opportunistic infections. Moreover, through mononuclear cell infection and activation, it directly causes many tissue damages such as the gut, brain, and lung. Apart from tissue injury, it damage body organs, including pulmonary, chronic cardiovascular, hepatic and central nervous system diseases, through chronic immune activation and endothelial dysfunction [64]. Although a cure for HIV does not yet exist, anti-retroviral therapy (ART) can improve the quality of life of HIV-infected people, although with considerable side-effects over time [66]. Considering this, as current ART regimens are life-extending but not curative, despite healthy resting CD4 counts and undetectable viral loads in HIV patients, it does not eradicate virus infection, which persists in a latent form even after prolonged treatment which is a major hurdle towards HIV eradication [66]. Exosomes, the subject of only recent investigation, have shown potential roles in multiple stages of HIV‐1 pathogenesis by transporting multiple cargoes to target cells, which will be described next [67, 68].

### Role of exosomes in HIV-1 infection

HIV-1 is the most widely recognized type of retroviruses among viruses [69]. HIV-related proteins can be transferred by exosomes to recipient cells and promote the spreading rate of infection by inducing recipient cells susceptible to HIV. In this regard, Nef, the HIV protein is transferred within exosomes [70, 71] and when these exosomes reach latent cells, activate the cells and make them more vulnerable to HIV infection [72]. Besides, Nef is capable of inhibiting the generation of CD4^+^ exosomes from T cells, consequently, suppresses viral recognition by immune cells [73]. Previous studies showed that exosomes bearing Nef induce death or senescence in CD4^+^ T lymphocytes [74, 75]. Scheafer and co-workers reported that macrophages release exosomes enriched with Nef that inhibits T cell function via beta-COP-dependent pathway and degradation of MHC-I and CD4^+^ molecules [76]. Moreover, Nef^+^ exosomes are capable of inhibiting the adaptive immune response by deterring the IgA and IgG production in B lymphocytes, accordingly, this event allows the virus to evade the humoral immune response [77].

Viral receptors carried by exosomes from HIV-1-infected cells can make target cells more vulnerable to infection. For example, exosomes produced by peripheral blood mononuclear cells (PBMNCs) and CCR5^+^ ovary cells convey CCR5 to CCR5 null cells, which enhances HIV-1 infection [78]. In support, a study by Rozmyslowicz et al*.* showed that exosomes produced by megakaryocytes and platelet are loaded by HIV co-receptors CXCR4, which disposes CXCR4-null cell to X4-HIV infection [79]. These data provide evidence that exosomes are implicated in transferring virus receptors and make recipient cells at risk of HIV infection in vitro; nonetheless, responses against animal models remain still unclear.

Besides, as mentioned above, viral nucleic acids can be loaded within exosomes, therefore these exosomes can increase infection rate via delivering viral genome to healthy cells. For example, exosomes from HIV-infected cells transmit the transactivation response element (TAR) RNA [80], which is placed at the 5′ tail of HIV transcript copies and interact with the Tat protein to produce viral RNAs. The TAR-RNA molecule generates miRNAs suppressing a Bcl-2 interacting protein, which ultimately promotes resistance to apoptosis and production of the virus [80, 81]. Indeed, TAR protects cells from apoptosis for the production of virus in infected cells.

On the other hand, exosomes from infected cells may function antiviral. For instance, exosomes from infected cells bear APOBEC3G molecules, the antiviral proteins prevent virus production via deamination of the cytosine residues to uracil in the minus strand of the viral DNA during reverse transcription. This feature is valuable, since the Vif, an opposite viral protein, is not sorted into exosomes [82]. Furthermore, cGAMP which is found in exosomes produced by infected cells can trigger antiviral response via innate immune responses and interferon upregulation [83, 84]. Bernard et al*.* demonstrated that HIV-related miRNAs like miRNA-88 and miRNA-99 induce endosomal NFkB and TLR8 signaling, which in downstream recalls immune response against HIV via TNF-α production from macrophages [85].

### Role of exosomes in HIV-1 mediated pro-inflammatory response

Inflammation is a pivotal factor involved in HIV infection [86]. Studies have shown that HIV proteins can activate pro-inflammatory cytokine signaling pathways. Pro-inflammatory cytokines are important for HIV pathogenesis as they play key roles in regulating the HIV lifecycle and T cells' apoptosis [86]. Exosomes have been reported to transfer viral pro-inflammatory components. For example, exosomes collected from CSF samples of HIV-positive individuals contain Tat and TAR proteins [87]. These viral components play a key role in inducing pro-inflammatory responses [88–90]. Mukhamedova et al*.* reported that Nef-containing exosomes can be taken up by macrophages and increase secretion of pro-inflammatory cytokines through ERK1/2 phosphorylation and activation of NLRP3 inflammasome [91]. Furthermore, Chettimada and colleagues characterized the protein cargo of exosomes from plasma isolated from ART-treated HIV patients and HIV-negative controls. In HIV^+^ patients, levels of plasma exosomes enriched with Notch4 and oxidative stress markers were elevated [92]. Treatment of THP-1 monocyte cells with these exosomes trigged pro-inflammatory responses during HIV pathogenesis via the overexpression of genes involved in interferon responses [92]. Aside from protein cargoes, Sampey et al*.* demonstrated that exosomes released by HIV-1-positive individuals carry an abundance volume of HIV-1 TAR-RNA and TAR miRNAs, which could have an important role in the HIV-1 pathological process. They showed that co-culturing of exosomes derived from HIV-1-infected cells with primary macrophages or mouse neuronal cells, stimulate secretion of IL-6 and TNF-α, the pro-inflammatory cytokines, through activating of NF-κB components by binding to PKR and/or potentially to TLRs [88]. In addition to vmiR-TAR, HIV vmiR88 and vmiR99 are present in exosomes secreted from HIV-infected macrophages and the sera of HIV^+^ individuals. These miRNAs, as ligands for TLR8 signaling, can induce an inflammatory response and may have a role as chronic immune activators [85]. Besides viral cargoes, Kulkarni et al*.* demonstrated that exosomes isolated from HIV-1 infected DCs contain fibronectin and galectin-3, which play a role in up-regulating the pro-inflammatory cytokines expression including IFN-γ, TNF-α, IL-1β, and RANTES as well as activation of p38/Stat pathway in T-cells [93]. These facts revealed that exosomes from infected cells induce inflammatory responses.

### Role of exosomes in HIV-associated neurological disorders (HANDs)

Neuronal cell injury and death are a hallmark feature of HANDs [94]. HAND demonstrates a comprehensive form of disorders that extent from moderate cognitive impairment to severe HIV-associated dementia (HAD) [95]. Despite the development of combined antiretroviral therapy (CART), the prevalence of moderate forms of neurocognitive impairment manifests remains high. Several studies hypothesized that the neurological abnormalities observed in HIV-infected individuals are associated with the amplification of HIV specific signals by unknown mechanisms [96]. There is evidence that one of these mechanisms could be mediated by exosomes. Rahimian et al*.* demonstrated that exosomes derived from Tat-expressing primary astrocytes transfer the biologically active HIV-1 Tat, which has the capacity of causing neurite reduction and neuron death and can be considered advantageous for HIV-1 pathogenicity [97]. Moreover, Khan et al*.* declared that plasma-derived exosomes from patients with HAD contain both Nef mRNA and Nef protein. They further demonstrated that Nef mRNA-containing exosomes can be uptake by SH-SY5Y neuroblastoma cells and translated into Nef protein, leading to beta-amyloid (Aβ) accumulation and secretion, which is involved in HAND progression [98]. Raymond et al*.* reported that exosomes derived from nef–gfp-transfected or HIV-infected microglia contain Nef protein. Exosomal Nef can disrupt blood–brain barrier permeability and integrity via reducing expression of the tight junction protein, ZO-1, and inducing inflammatory cytokines expression, so maybe play a significant role in HIV-induced neuropathogenesis [99]. Interestingly, Hu et al*.* demonstrated the increased levels of miRNA-29b in the basal ganglia in the brains of the morphine-dependent simian immunodeficiency virus (SIV)-infected rhesus macaques. They further demonstrated that miRNA-29b is present in exosomes isolated from astrocytes treated with both morphine and HIV Tat, which can interact with neurons, results in the downregulation of PDGF-B expression and neuronal cell death [94].

### Role of exosomes and HIV-associated malignancy (HAM)

People living with HIV/AIDS may be at higher risk of cancer, which is known as HIV-associated malignancy (HAM) [100]. Traditionally, HAM is categorized into two types: AIDS-defining cancers (ADCs) and non-AIDS-defining cancers (NADCs). Primary central nervous system lymphoma, cervical cancer, non-Hodgkin's lymphoma, and Kaposi sarcoma are classified as ADCs and others such as lung, liver, anal, and melanoma NADCs [101]. Although the underlying mechanism deals with the development and progression of HAM is not yet completely understood, the association between exosomes secreted from HIV-infected cells and HAM has attracted attention in recent years. Li et al*.* investigated that exosomes from HIV-infected T lymphocytes contain miRNA-155-5p which can contribute to the advancement of cervical cancer cells via the ARID2-ERCC5-NF-κB signaling pathway and up-regulating the expression of such cytokines as IL-1, IL-6, and IL-8 [100]. Besides, Chen et al*.* reported that TAR RNA-containing exosomes secreted from HIV-infected T-cells, by activation of the ERK1/2 signaling, may have a potential role in the development of lung cancer and HNSCC [102].

### Application of exosomes in HIV-1 associated diseases

#### HIV-1 exosome-based vaccines

Despite the advantages of ART in improving the life quality of patients with HIV infection, there are several limitations and adverse effects regarding this combination of therapies, so the development of alternative therapeutics or efficient vaccines is necessary for HIV-1 infected patients [103]. Exosomes as biological nano-vesicles with a low immunogenic profile, hold much promise for developing a new nanoparticle-based vaccine [104]. Lattanzi et al*.* proposed a novel exosome-based CD8^+^ T cell vaccine that depends on the features of an inactivated mutant form of the HIV-1 Nef protein (Nef^mut^). They compared the immunochemical properties of Nef^mut^ exosomes with Nef^mut^-based lentiviral virus-like particles (VLPs) and indicated that the plan of HIV-1 Nef^mut^ -based exosome vaccines could correctly surmount main problems regarding safety, production and isolation mostly met by vaccine development based on chimeric VLPs. The reality that Nef^mut^-based exosome development do not be composed of further lenti- or retroviral products and are lack of anti-cellular effects open up new horizons on the way to the development of still-unproven vaccine strategies [105]. Nanjundappa et al*.* generated HIV-1 Gp120-specific T cell-based Gp120-Texo vaccine by ConA-stimulated C57BL/6 (B6) mouse CD8^+^ T (ConA-T) cells with the uptake of pcDNAGp120-transfected B6 mouse DC line DC2.4 (DC2.4Gp120)-released exosomes and indicated that CD8^+^ Gp120-Texo vaccine resulted in protective and durable immunity against Gp120-expressing B16 melanoma in both wild-type C57BL/6 and transgenic HLA-A2 mice which may indicate a modern vaccine for the immunotherapy of immunocompromised patients with HIV-1 infection [103, 106]. Since HIV Gag is considered as one of the most major and important antigen candidates for the development of HIV-1 vaccine, Wang et al*.* generated HIV-1 Gag-specific Gag-Texo vaccine using ConA-stimulated polyclonal CD8^+^ T-cells with the uptake of Gag-expressing adenoviral vector AdV_Gag_-transfected DC released exosomes. They investigated the stimulation of Gag-specific CD8^+^ CTL responses and antitumor immunity. Gag-Texo-stimulated CTL responses, which resulted in defensive immunity against Gag-expressing BL6-10_n_ melanoma in wild-type C57BL/6 and transgenic HLA-A2 mice and may stand for a novel immunotherapy vaccine for HIV-1 patients [107].

### Exosomes as biomarkers for HIV-1

Recognition of specific exosomal proteins or RNAs in plasma EVs from HIV^+^ patients could serve as a diagnostic purpose for HIV infection [108]. The evaluation of specific miRNAs in EVs holds great promise in this respect. Exosomes obtained from a subgroup of HIV-1-infected elite controllers demonstrated that miRNA-21 was down-regulated in HIV controlled patients, with a decreasing sequence of T CD4 lymphocytes, so authors suggested exosome-derived miRNA-21 as a possible valuable predictive biomarker in HIV-1-infected elite controllers patients [109]. HNAD, described by Aβ deposition, is particularly frequent in people with HIV-1, which already is a conventional biomarker for Alzheimer’s disease [110]. Sun et al*.* demonstrated that neuron-derived exosomes (NDE) from HIV-positive and negative individuals indicated that neuropsychiatric disabilities diminish the whole amount of NDEs and augment them with high-mobility group box 1 (HMGB1), neurofilament-light (NF-L), and Aβ. These investigations propose the possible function of NDEs in the role of cognitive damage biomarkers in HIV-1 infected subjects [111]. Besides, EVs are appropriate candidate to be applied as biomarkers in HIV-infected drug abusers [112]. In this regard, to investigate the role of alcohol and tobacco, which are frequent in HIV positive patients on the exosomal cytokines and chemokines, Kodidela et al*.* assayed the levels of cytokines and chemokines in the plasma and plasma exosomes of HIV-infected individuals in different groups [113]. They indicated that IL-8 and IL-10 were higher respectively in the HIV-positive alcohol drinkers and HIV-positive smokers, in comparison to the non-drinkers and non-smokers HIV-positive cases. Their findings suggested that alcohol and tobacco usage altered the exosomal cytokines and chemokines contents and may have substantial roles in toxicity and disease progression in HIV-positive drug abusers [113]. Subsequently, this group performed a proteomic assessment for exosomes obtained from the plasma of HIV infected cases, who routinely used alcohol or tobacco, or both. They found different levels of hemopexin, alpha-2-macroglobulin, and properdin in the exosomes of the HIV-positive drinker and smoker groups. These altered exosomal proteins could serve as biomarkers for HIV-positive patients who abuse drugs [114].

### Exosomes as a therapeutic tool for HIV-1

Multiple studies have shown that exosomes from different origins may have anti-HIV bioactivity [115]. In this regard, Madison et al. described the antiretroviral role of healthy human semen exosomes that can block the transmission of HIV-1 by disturbing the viral replication in the vaginal epithelial cells which may be exploited for the development of novel anti-HIV therapeutic strategies [116]. Naslund et al. observed that healthy human breast milk-derived exosomes inhibit the HIV-1 infection of monocyte-derived DCs, via competitive binding to the DC-SIGN receptor [117]. In another study, Tumne et al. demonstrated that exosomes derived from CD8 + T cells, elicit a non-cytotoxic suppressive effect on HIV replication, due to suppression of the HIV-1 LTR promoter transcription [118].

Besides studies for the discovery of new exosome origins with congenital activity against HIV, research should focus on modified exosomes loaded with anti-HIV RNA with target peptides on their surface to achieve the highly effective delivery of RNA therapeutics to cells infected with HIV or a safe adjuvant treatment [115].

### Exosomes and human T-cell leukemia virus (HTLV)-1

HTLV-1, discovered in the early 1980s, was the first retrovirus identified from humans [119]. Despite affecting up to 10 million people globally, HTLV-1 infection is considered a neglected disease nowdays [120]. HTLV-1 is classified as a Deltaretrovirus genus. The viral genome is consists of specific genes such as gag, pro, pol, and env, which encode retroviral proteins [120]. The transmission routes of HTLV-1 include sexual activity, transfusion of infected blood products, and maternal breast-feeding [120]. HTLV-1 can induce excessive clonal proliferation of CD4^+^ T cells and may result in cancer called Adult T Cell Leukemia-Lymphoma (ATLL) that has four clinical subtypes: smoldering, chronic, acute, and lymphoma [121]. Two regulatory proteins of HTLV-1, Tax [122] and Rex [123], as well as HTLV-1 Basic Zipper Protein (HBZ) [124], are involved in the development of ATLL [123]. Moreover, HTLV-1 causes a chronic inflammatory disease of the CNS known as Tropical Spastic Paraparesis/ HTLV-1 Associated Myelopathy (TSP/HAM) [125]. Nevertheless, HTLV-1 is associated with inflammatory diseases such as uveitis, infective dermatitis, and arthritis [119]. Although HTLV-1 has been discovered for 40 years, there is even disagreement about the mechanisms by which HTLV-1 may expand viral spread or prognosis for HTLV-1 diseases [126]. The current treatment options for people diagnosed with HTLV-1-associated malignancy or neuropathy are non-specific based on classical anti-cancer and anti-HIV medications [127]. Moreover, novel therapeutic advances in particular monoclonal antibodies have yielded hopeful outcomes and exhibited remarkable cytotoxic impacts on ATLL cells [127]. Despite multiple studies regarding the implication of exosomes in viral pathogenesis especially HIV-1, there have been only limited studies considering the roles of exosomes in HTLV-1 infection, and the first study was published in 2014 [128].

### Role of exosomes in HTLV- 1 infection

There is now evidence that HTLV-1 uses the exosomal secretory pathway for spreading and infection. For example, Alefantis et al*.* reported that Tax which is a trans-activating protein is secreted within exosomes from infected cells [129]. This protein is involved in the immune dysregulation and sorted into exosomes of infected cells through ESCRT-dependent machinery [128, 130]. Besides, HTLV-1-infected cells release exosomes containing viral mRNA and miRNAs including tax and hbz [128], which are implicated in infection progression and impairment of autophagy in healthy cells [126]. In another proteomics and genomics study, it was shown that exosomes from infected cells contain viral proteins such as gp46 and Tax, as well as cytokines like IL-10 and IL-6; and mRNA transcripts of Env, HBZ, and Tax [131]. Similar to HIV infected cells, exosomes produced by HTLV-1-infected cells contain distinct cargo as compared to those derived from uninfected cells, indicating regulation of host cell [54].

Exosomes from infected cells may also negatively regulate HTLV-1 infection. Tax mRNA and protein have been reported to induce the production of proinflammatory cytokines such as IL-6 and TNF-α in recipient microglia cells [132]. Exosomes transferring Tax protein have the potential to increase the survival rate of cytotoxic T cells and uninfected peripheral blood mononuclear cells (PBMCs) in culture in an IL-2 dependent manner [128]. Besides, exosomes from HTLV-1-infected cells contain Tax protein that sensitizes recipient cells for lysis by T lymphocytes [133]. Exosomes-associated Tax can be purified from HTLV-I associated neurologic disease (HAM/TSP) patients PBMCs and CSF samples [126]. It was reported that exosomes derived from PBMCs of HAM/TSP lessen the CD4^+^CD25^+^ T cells when co-cultured with uninfected PBMCs [133]. This fact supports the idea that exosomes containing HTLV-1 antigen may promote inflammatory responses in HAM/TSP via suppressing regulatory T cells (Tregs) function [134–136]. Altogether, exosomes from infected cells contain HTLV-1 components that promote infection, however, some colleagues have indicated that these exosomes may negatively regulate HTLV-1 infection via inducing immune cell responses [137, 138]. Further research must be undertaken to elucidate the exact role of exosomes in HTLV-1 related disease and the possible targeting of therapeutics.

## Conclusion

Exosomes derived from human retroviruses-infected cells have been shown to perform a pivotal function in the pathogenesis of retroviruses (HIV-1 and HTLV-1), promoting infection and inflammation responses. These exosomes contain various types of virus-related nucleic acid and proteins that trigger functional changes in recipient cells. Exosomes may serve as a potential tool for anti-viral therapies such as exosome-based vaccines and biomarkers of retroviruses-associated diseases. However, future studies on the current topic are therefore required to elucidate the exact role of exosomes in virus infection and to determine the clinical application of these exosomes for preventing infection.

## Data Availability

Not applicable.

## Abbreviations

AIDS: Acquired immunodeficiency syndrome
ADCs: AIDS-defining cancers
ALIX: ALG-2 interacting protein
ART: Anti-retroviral therapy
ATLL: Adult T Cell Leukemia-Lymphoma
CSF: Cerebrospinal fluid
DC: Dendritic Cells
ESCRT: Endosomal Sorting Complex Required for Transport
EVs: Extracellular Vesicles
FBS: Fetal bovine serum
HAM: HIV-associated malignancy
HAM/TSP: HTLV-I associated neurologic disease
HANDs: HIV-associated neurological disorders
HBZ: HTLV-1 Basic Zipper Protein
HIV-1: Human Immunodeficiency Virus -type 1
HMGB1: High-mobility group box 1
HSC70: Heat shock cognate 71 kDa protein
HSP70: Heat shock 70 kDa protein
HTLV-1: Human T-cell leukemia virus-1
ICAMs: Intercellular adhesion molecules
ILVs: Intraluminal Vesicles
ISEV: International Society of Extracellular Vesicles
LIMK: LIM kinase
MVB: Multivescular body
MVs: Microvesicles
MYLK: Myosin Light Chain Kinase
NADCs: Non-AIDS-defining cancers
NDE: Neuron-derived exosomes
NF-L: Neurofilament-light
PBMNCs: Peripheral Blood Mononuclear Cells
ROCK: Rho-associated protein kinases
TAR: Transactivation response element
Tregs: Regulatory T cells
TSP/HAM: Tropical Spastic Paraparesis/ HTLV-1 Associated Myelopathy
